# Structural, Magnetic, and Electron Spin Resonance Properties of Nickel Nanoferrites Synthesized by High‐Energy Ball Milling

**DOI:** 10.1002/open.202500463

**Published:** 2026-01-26

**Authors:** Sanele Dlamini, Sizwe Masuku, Mohd Sajid Ali, Hamad A. Al‐Lohedan, Gulam Rabbani, Lebogang Kotsedi, Teboho Mokoena, Tebogo Mahule, Teboho Mokhena, Mohd. Hashim, Justice Msomi, Amos Nhlapo

**Affiliations:** ^1^ School of Chemicals and Physical Sciences Faculty of Agriculture and Natural Science University of Mpumalanga Mbombela South Africa; ^2^ Department of Chemical and Physical Sciences Walter Sisulu University Mthatha South Africa; ^3^ Department of Chemistry College of Science King Saud University Riyadh Saudi Arabia; ^4^ School of Chemical Engineering Yeungnam University Gyeongsan Republic of Korea; ^5^ iThemba LABS‐National Research Foundation Somerset West South Africa; ^6^ Department of Physics University of the Free State Phuthaditjhaba South Africa; ^7^ Department of Physics University of South Africa Johannesburg South Africa; ^8^ DSI/Mintek‐Nanotechnology Innovation Centre Advanced Materials Mintek Randburg South Africa; ^9^ Department of Applied Physics Aligarh Muslim University Aligarh India; ^10^ Department of Medical Physics Sefako Makgatho Health Sciences University Medunsa South Africa

**Keywords:** ball milling, Electron Spin Resonance, magnetization, NiFe_2_O_4_ ferrite, Spinel structure

## Abstract

Nanocrystalline NiFe_2_O_4_ was synthesized using high‐energy ball milling. The effect of milling time on structural and magnetic properties was investigated. X‐ray diffraction results revealed a progressive transformation from mixed NiO–Fe_2_O_3_ precursor phases to a single‐phase cubic spinel NiFe_2_O_4_ structure with crystallite sizes ranging from 33.64 to 41.17 nm. The scanning electron microscopy showed small grains attaching to big grains for 1 h milled sample. The big grains disappear with increasing milling time. Homogeneous nanoparticles, spherically shaped and agglomerated nanoparticles, were observed for samples that were milled for 5, 10, and 15 h. Energy‐dispersive X‐ray spectroscopy confirmed the presence of all expected elements. The nature of *M* *–* *H* loops for all the samples shows soft ferromagnetic behavior. The Electron spin resonace (ESR) results revealed the reduction of resonance field with increasing milling time. The *g*‐values increased with milling time. The obtained high *g*‐values make NiFe_2_O_4_ oxides suitable for applications in high‐frequency devices. The spin–spin (*τ*_1_) relaxation time decreased with increasing milling, time while the spin–lattice (*τ*_2_) showed improvement.

## Graphical abstract

One of the properties of nickel ferrite materials that is drawing attention is their potential for biomedical applications. In this work, NiFe_2_O_4_ nanoparticles from NiO and Fe_2_O_3_ were produced using high‐energy ball milling. Milling time enhanced crystallinity. Magnetization tests and ESR studies revealed soft ferromagnetic materials, emphasizing their importance in biomedical applications.

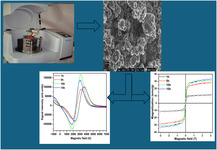

## 1 Introduction

Ferrite is a ceramic material formed by grinding the needed amounts of iron (III) oxide (Fe_2_O_3_) with small amounts of one or more other metallic elements, such as Ni, Mn, Zn, Ba, Cu, Sr, etc. Ferrites typically have a spinel structure with the formula AB_2_O_4_, wherein A and B are various metal cations, frequently incorporating iron (Fe) [1, 2].

In routinely used FCC oxides (O^2−^), A cations occupy one‐eighth of the tetrahedral holes while B cations fill half of the octahedral holes. Nanosized nickel ferrite (NiFe_2_O_4_) is a delicate magnetic ferrite that has low saturation magnetization (*M*_s_) and coercivity (*H*_c_). NiFe_2_O_4_ has an inverse spinel structure, which is face‐centered cubic (FCC) [3]. Nickel ferrite's unit cell consists of 32O^2−^, 8Ni^2+^, and 16Fe^3+^ ions. Oxygen ions form 64 tetrahedral (A) and 32 octahedral (B) sites, each of which holds 24 cations. In the inverse spinel structure, Fe^3+^ ions occupy the whole A site, whereas Ni^2+^ and Fe^3+^ ions segment the B site. From this, the ferrimagnetism in NiFe_2_O_4_ is a consequence of antiparallel spins between Fe^3+^ and Ni^2+^ at A and B sites, respectively.

The recent and ongoing development and use of electronic devices in our daily lives has raised numerous concerns about electromagnetic interference [4, 5, 6]. Ferrite nanoparticles with spinel structures are known for their high surface area, enhanced physical, chemical, and magnetic properties, making them suitable for a wide range of applications in fields such as gas sensing, catalysis, electronics, and biomedical diagnosis [7, 8, 9]. Various parameters, including cation dispersion, crystallite size, manufacturing technique, and type of dopents , make them easily tunable. Nickel ferrites have been shown to be suitable for a variety of applications, including photocatalysis and biomedicine [10]. Nickel ferrites have distinct properties, such as high adsorption capacity, remarkable stability, safety, good magnetic properties, and electronic tunability, which make them an excellent candidate for the removal of environmental pollutants due to their adsorption properties. They are also utilized as sensors for detecting trace contaminants at low concentrations, with good sensitivity and selectivity [11].

Previous findings have shown that milling causes structural evolution. Bid et al. [12] produced NiFe_2_O_4_ using ball milling and investigated the effect of milling on the microstructure characterization of mechano‐synthesized nanocrystalline NiFe_2_O_4_ using Rietveld's analysis. Priyadarshini et al. [1] produced NiFe_2_O_4_ via coprecipitation and examined the impact of annealing on structural and magnetic characteristics. The authors reported that annealing temperature increased saturation magnetization and *g*‐values. Zang et al. [13] produced NiFe_2_O_4_ using a planetary ball milling‐assisted solid‐state reaction. They reported improved magnetic properties following annealing. Hajalilou et al. [14] investigated nickel ferrite nanoparticles produced by high‐energy planetary ball milling of a stoichiometrically combined NiO and Fe_2_O_3_ powders. The NiFe_2_O_4_ nanoparticles, generated by the diffusion and counter‐diffusion of Ni^2+^ and Fe^3+^ ions in the mechanochemical reaction, exhibited ferromagnetic behavior with a magnetization saturation of around 8.5 emu/g and negligible coercivity. Morphology is one of the preliminary factors that influences magnetic properties. Thus, researchers have attempted to synthesize magnetic nanoparticles with smaller particle sizes and uniform morphology.

Recently, researchers have used numerous and different synthesis methods, such as combustion [15], coprecipitation [16], sol–gel [17], microwave assisted [18], ball milling [19], solid state reaction [20], and hydrothermal [21], amongst others to fabricate single‐phase spinel ferrite materials. In recent years, high‐energy milling has become a popular technique. The milling process's high nonequilibrium character allows for the creation of solids with improved and/or unique physical and chemical properties. High‐energy milling using high‐purity NiFe_2_O_4_ powders in a planetary ball mill at room temperature yielded a stable nanocrystalline nickel ferrite with a crystallite size of about 9 nm [22].

To the best of our knowledge, only a few papers have examined the effect of milling duration on the crystal structure, shape, and magnetic properties of NiFe_2_O_4_ nanoparticles. This integrated structure–magnetism–ESR correlation is not reported in the cited literature. In this work, we examined the impact of milling time on the structural, magnetic, and electron spin resonance properties of NiFe_2_O_4_ nanoparticles generated via high‐energy ball milling. All the samples were subjected to powder X‐ray diffraction, scanning electron microscopy coupled with EDX, vibrating sample magnetometer, and electron spin response.

## 2 Experimental Details

### 2.1 Materials and Synthesis

Nickel ferrites (NiFe_2_O_4_) compounds were produced from solid state method using high‐energy ball milling as previously described [23]. High‐purity Fe_2_O_3_ (99.9%) and NiO (99.9%) metal oxides, supplied by Sigma Aldrich Company of South Africa, were used as starting materials. For a 5 g of NiFe_2_O_4_ sample, 3.3121 g of Fe_2_O_3_ and 1.5774 g of NiO were weighed and mixed by mechanical milling using a Retch planetary ball mill (type: PM 400) operated at 300 rev/min. The milling medium consisted of a hardened‐steel vial filled with hardened‐steel balls with a diameter of 13.4 mm. The total mass of the powder was 5 g, and the ball‐to‐powder mass ratio was fixed to 10:1 under an environment of air without any additive. 1 g of sample was removed from the mixture after the time intervals of 1, 5, 10, and 15 h. The corresponding 10 g of ball was also removed. To improve crystallinity by reducing defects and the organic impurities, all extracted sample powders were annealed at 800°C for 4 h. The final products were then taken for different characterization techniques.

### 2.2 Characterization Techniques

A D2 phaser diffractometer was used to acquire X‐ray diffraction (XRD) patterns. The Zeiss Supra 55 VP scanning electron microscopy (SEM) coupled with Energy‐dispersive X‐ray spectroscopy (EDS) spectroscopy was used to investigate the morphology of the nanoparticles. EDS was used to study and confirm the elemental compositions. The room temperature hysteresis loops were measured using a vibrating sample magnetometer (VSM) from Cryogenic Ltd. in the United Kingdom. The ESR properties were measured at room temperature on a Bruker EMX spectrometer operating at 9.45 GHz.

## 3 Results and Discussions

### 3.1 XRD Results

**FIGURE 1:**
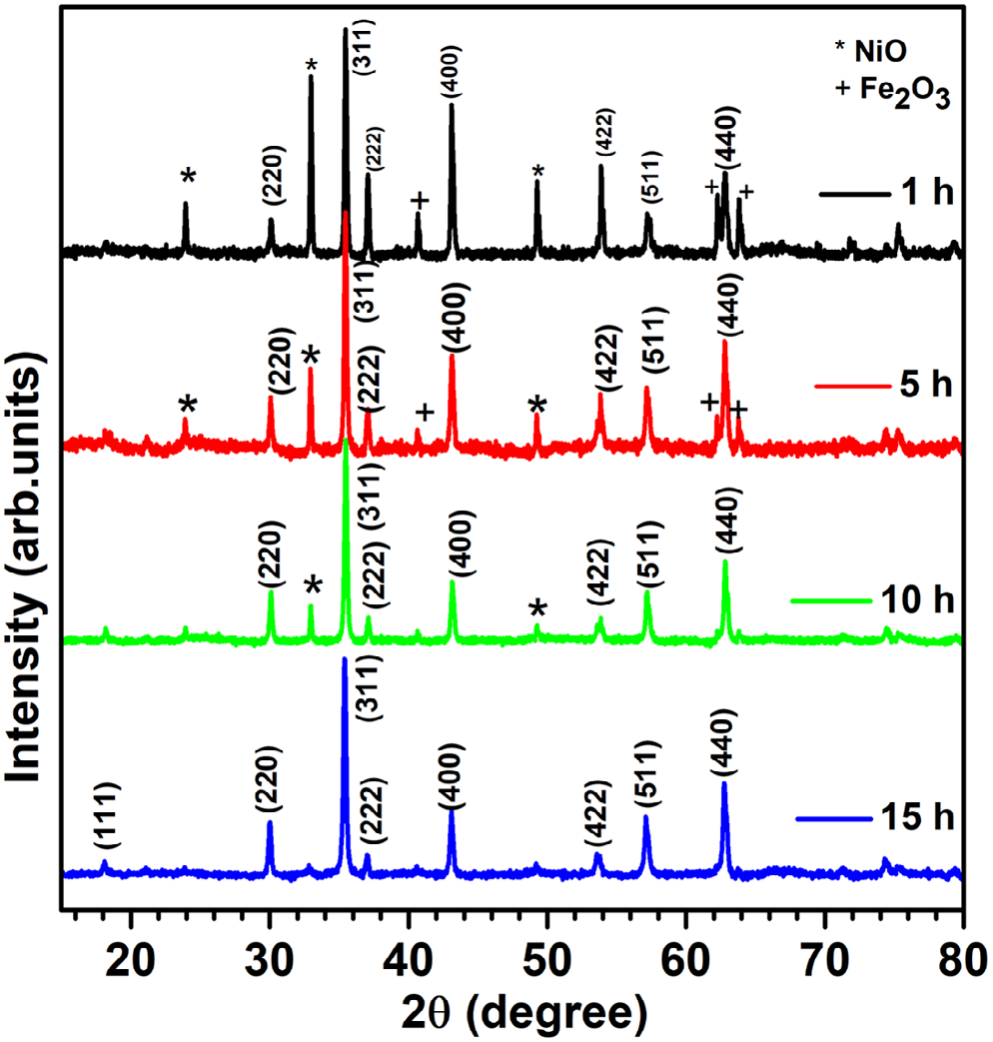
XRD spectra for NiFe_2_O_4_ nanoferrites.

**FIGURE 2:**
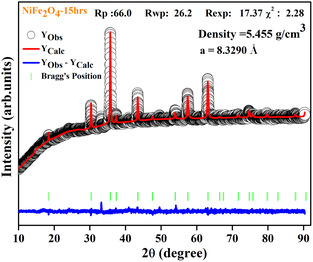
XRD pattern of refinement using Full‐prof software for the 15 h milled NiFe_2_O_4_ sample.

The structural examination of the nickel ferrite nanoparticles was investigated by the XRD, as depicted in Figure 1. The observed XRD patterns showed impurities associated with unreacted precursors of Fe_2_O_3_ and NiO oxides at lower milling times. The presence of these impurities in XRD patterns gives conclusive evidence that extensive milling is required for the thorough reaction crystallization of the Nickel ferrite phase. With increasing milling time, a gradual decrease in impurities was observed, which correlates with the structural transformation from mixed‐phase structure to a single‐phase cubic spinel structure. Almost similar behavior was observed for NiFe_2_O_4_ ferrites synthesized by ball milling [13, 14]. After 15 h of milling, the XRD pattern of NiFe_2_O_4_ nanopowders shows characteristic reflections of (111), (220), (311), (222), (400), (422), (511), (440), and (533) planes, confirming the formation of face‐centered cubic structure of nickel ferrite. The strong and crisp reflection peaks indicate that nanoparticles have a high degree of crystallinity. All peaks match well with the normal CPDS card no. 74‐2081 [24], No secondary peaks are detected in XRD pattern which ensures the phase purity. Structural parameters, such as crystallite size, lattice parameters, volume of the unit cell, X‐ray density, and specific surface area for NiFe_2_O_4_ nanoferrites are tabulated in Table 1. Figure 2 depicts the Rietveld analysis for the 15 h milled NiFe_2_O_4_ sample. In this figure, the black circle symbol shows the observed XRD pattern, the red line represents the calculated Rietveld refined pattern, and the blue line shows the difference between the XRD and the Rietveld refined pattern of the nickel ferrite nanoparticles. Green bars indicate the Bragg's location for the nickel ferrite nanoparticles. The observed peaks indicate a single phased inverse spinel structure space group $Fd\bar{3}m$ of NiFe_2_O_4._

**TABLE 1:** Crystallite size (*D*), lattice parameters (*a*), dislocation density (*δ*), volume of the unit cell (*V*), X‐ray density (*ρ*), and specific surface area (*SSA*) for NiFe_2_O_4_ nanoferrites.

Milling time, h	*D*_XRD_, nm	*δ* × 10^−4^, nm^*−2*^	*a,* Å	*V,* Å^3^	*ρ,* g/cm^3^	*SSA,* m^2^/g
1	41.16	5.903	8.3851	589.6	5.2730	27.65
5	37.53	7.099	8.3885	590.3	5.2707	30.33
10	35.85	7.781	8.3836	589.2	5.2685	31.77
15	33.63	8.842	8.4006	592.8	5.2525	33.97

**FIGURE 3:**
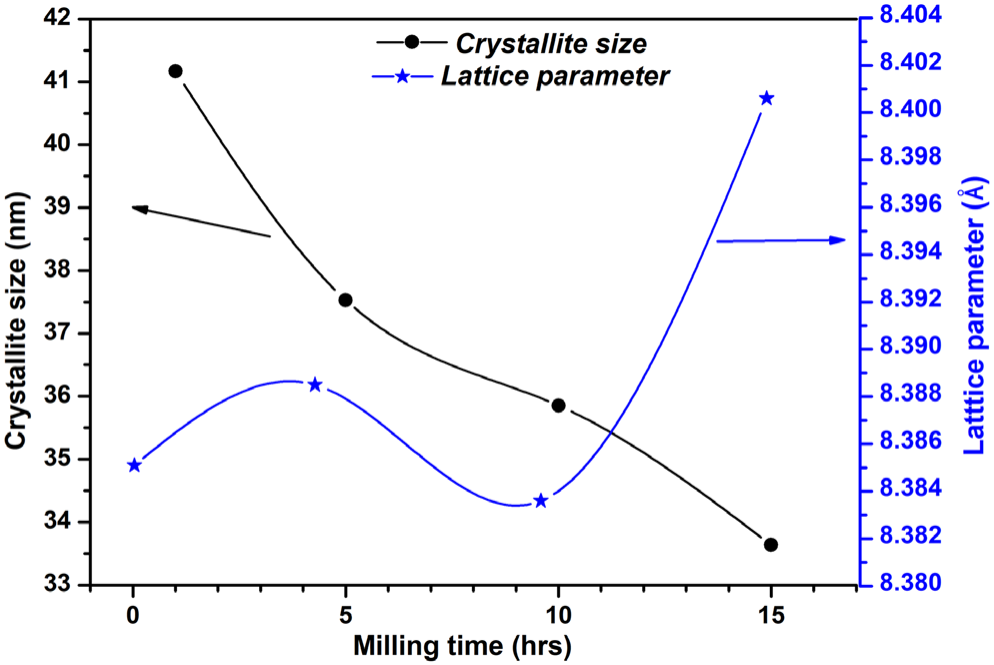
Variation of crystallite sizes and lattice parameter as function of milling time for NiFe_2_O_4_ nanoferrites.

The crystallites sizes compounds were estimated using Debye–Scherrer formular [25]:

(1) $$D=\frac{0.91\lambda }{\beta \cos\theta }$$

where *λ* denotes the X‐ray wavelength, *β* denotes the full‐width at half maximum of the diffraction peak, while *θ* is the Braggs’ angle [26]. The obtained crystallite sizes were 41.53 nm for 1 h milled, 37.53 nm for 5 h, 35.85 nm for 10 h, and 33.64 nm for the 15 h milled sample. Figure 3 reveals the general decrease in crystallite size with milling time. Milling has as significant impact on crystallite sizes. Similar observation were reported previously [23, 27, 28].

The dislocation density (*δ*) in a crystal was determined using the expression [29]:

(2) $$\delta =\frac{1}{D^{2}}$$

From Table 1, the dislocation values increase from 5.9035 × 10^−4^ at 1 h milled sample to 8.842 × 10^−4^ at 15 h milled sample. The increase in dislocation with milling is related with the increase in interplanar d‐spacing. The dislocation raises with the increasing grains [29].

The lattice parameters (*a*) of the compounds were estimates using the Bragg's law [30]:

(3) $$a=d_{hkl}\sqrt{\left(h^{2}+k^{2}+l^{2}\right)}$$

where *a* is the lattice parameter, *d* is the inter‐planar spacing, and *h*, *k,* and *l* are the Miller indices of the crystal planes*.* The inter‐planar spacing (*d*) was calculated by using the well‐known Bragg law of XRD [31]:

(4) nλ=2dsinθ

where *n* is the order of diffraction, and other symbols have usual meanings. In contrast to crystallite sizes, the obtained lattice parameters showed a general increase with milling time, as observed from Figure 3. The slight decrease between 5 and 10 h of milling is attributed to the structural evolution.

The total surface area (SSA) per unit mass of these materials was estimated using the relation [32]:

(5) $$SSA=\frac{6000}{D_{\mathrm{XRD}}\rho_{x}}$$

*D*_XRD_ denotes grain sizes (m) while *ρ*_*x*_ denotes the X‐ray density (g/m^3^). The variation of *SSA* is attributed to changes in crystallite sizes and X‐ray densities.

### 3.2 SEM and EDS Results

**FIGURE 4:**
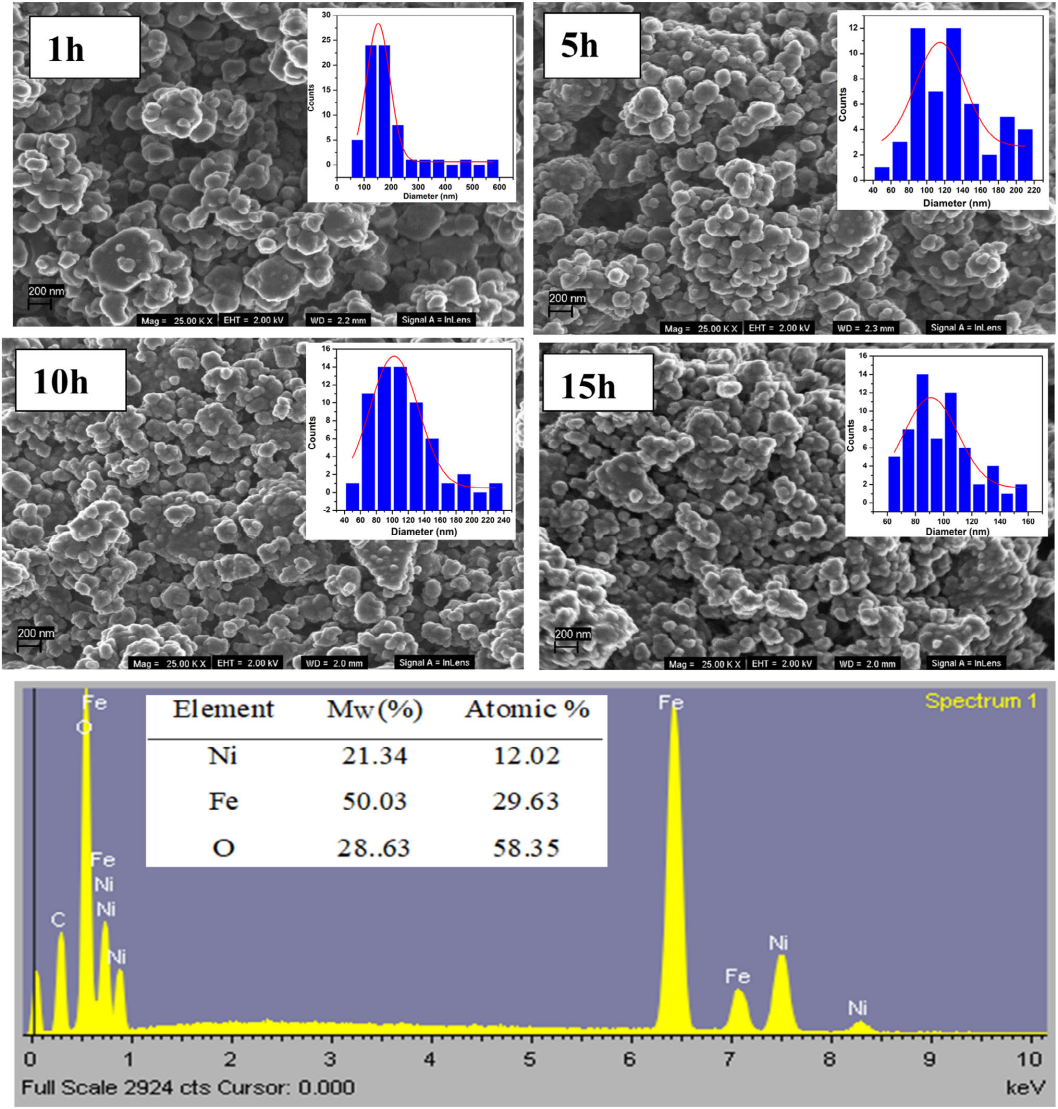
SEM images spectra as a function of milling time, and a typical EDS spectrum with inserted elemental composition, for NiFe_2_O_4_ nanoferrites.

The morphology of NiFe_2_O_4_ ferrite nanopowders synthesized by ball milling was investigated using scanning electron microscopy (SEM). Samples were coated with a carbon coating to about 40 nm thickness. The SEM micrographs of NiFe_2_O_4_ nanoparticles with different milling times are presented in Figure 4. The images for 1 h milled sample show bulgy particles and small particles attaching or clamping to them. The clamping is associated with magnetic nature of the particles. These bulgy particles disappear as the milling time increases. Samples milled for 5, 10, and 15 h reveal evenly distributed, spherical‐form and slightly agglomerated nanoparticles. For all samples, the expected elements are the same, therefore a representative EDS spectrum for the NiFe_2_O_4_ oxides generated by ball milling for 1 h is also displayed in Figure 4. During the measurements, excess of 300,000 X‐rays was detected when an acceleration voltage of 20 kV was applied. All expected elements such as nickel (Ni), iron (Fe), and oxygen (O), were detected. The observed extra carbon (C) peak is due to fluorescence and artifact during grid coating. No other unexpected or impurity peaks were detected. This confirms the successful synthesis of pure NiFe_2_O_4_ ferrites by ball milling. The average of the experimentally obtained molecular weight (*M*_W_) and atomic weight percentages are inserted on the EDS graphs.

### 3.3 Magnetization Results

**FIGURE 5:**
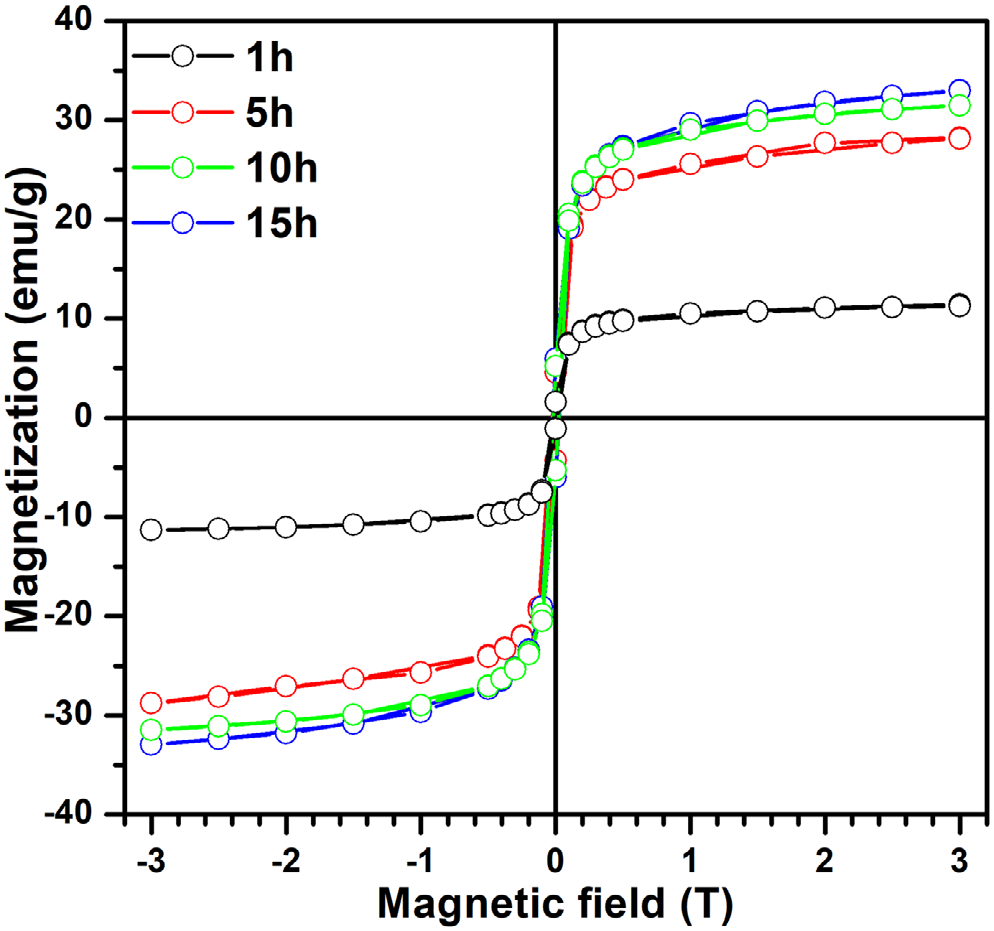
Room temperature magnetic hysteresis loop of NiFe_2_O_4_ nanoferrites for various milling hours.

The magnetization (*M–H*) curves of NiFe_2_O_4_ samples synthesized via high‐energy ball milling for various durations (1, 5, 10, and 15 h) are shown in Figure 5, measured at 300 K under an applied magnetic field between −3 T and 3 T. The “S” shaped magnetization curves with small coercive fields, samples demonstrate soft ferrimagnetism with features approaching superparamagnetic‐like behavior. The nature of the *M–H* loops for all the samples shows soft ferromagnetic behavior. The shape arises from reduced domain‐wall pinning due to smaller crystallite sizes, improved cation redistribution, and enhanced surface‐spin effects. The values of the saturation magnetization were determined by estimation of the Law of Approach to Saturation (LAS) using the formula [33, 34, 35]:

(6) $$M\left(H\right)=M_{S}\left(0\right)\left[1-\frac{a}{H}-\frac{b}{H^{2}}\right]+\chi H$$

**FIGURE 6:**
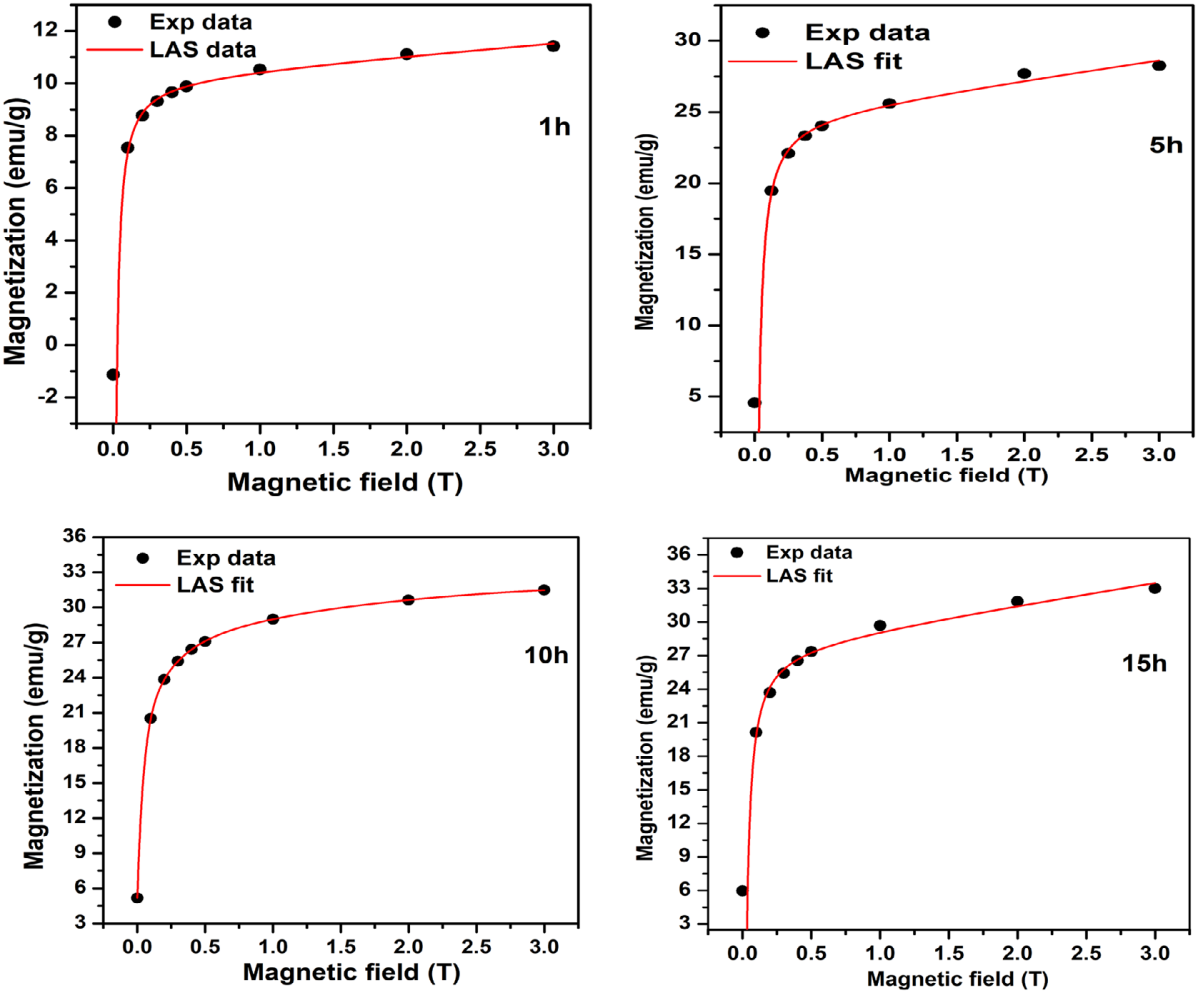
Initial magnetizations with best fit curves applying the empirical LAS magnetization for of NiFe_2_O_4_ nanoferrites for various milling hours.

In this eformular, *a* and *b* are fitting parameters, *H* denotes the applied magnetic field, and *χ* is the high‐field susceptibility. Figure 6 presents the obtained fitings for 300 K measurements. The values obtained are tabulated in Table 2 with other magnetic properties such as anisotropy, where *b* is a fitting constant, domain wall energy, and magnetic moments per unit formula. An increase in *M*_s_ was observed with milling time, rising from 10.23 emu/g at 1 h to 31.32 emu/g at 15 h. This trend correlates with structural transformation, evidenced by XRD patterns, where the presence of secondary hematite phases (*α*‐Fe_2_O_3_) in the early milling stages was suppressed and eventually eliminated with prolonged milling. Similar behavior was also observed in other studies in the literature [36, 37, 38]: The enhanced magnetization is attributed to improved cation redistribution within the spinel lattice, as suggested by the Goodenough–Kanamori rules, whereby Fe^3+^ ions preferentially occupy tetrahedral (A) sites and Ni^2+^ ions are stabilized at octahedral (B) sites, reinforcing A–B superexchange interactions [39]. In addition, this process is attributed to the greater redistribution and reordering of the cations in the spinel lattice that leads to a more desirable distribution of Fe^3+^ at A‐sites, and Ni^2+^ at B‐sites to enhance A–B superexchange (Fe^3+^(A)‐O^2‐^Fe^3+^/Ni^2+^(B)) [39]. This prevailing tendency can also be attributed to the decrease of crystallite size (41.16 to 33.63 nm), the increase in microstrain (5.903 × 10^−4^ to 8.842 × 10^−4^ nm^−2^), and the increase in surface area, which, coupled with each other, increases the surface spin alignment and inter‐grain coupling.

**TABLE 2:** Magnetic parameters of NiFe_2_O_4_ nanoferrites at 300 K showing the effect of milling time on saturation magnetization from LAS (*M*_s_), anisotropy constant (*K*_1_), domain wall energy, and magnetic moment per formula unit (*n*_B_).

Milling Time, h	*M*_s_, emu/g	*H*_C_, Oe	*K*_1_ × 10−2, J/m^3^	Domain Wall Energy, J/m^2^	*η*_B_, *μ*_B_
1 h	10.23	206	0.953	1.1169	0.4802
5 h	24.84	229	4.214	5.8523	1.1964
10 h	27.89	236	4.388	6.6089	1.3241
15 h	31.32	244	5.317	7.6250	1.3877

These structural properties are postulated in the surface spin theory and finite size influences to strengthen the surface spin alignment and interparticle exchange terms, which would increase the net magnetization [40]. These theories facilitate the *S*‐curves results that manifest when the magnetic hysteresis loop takes the form of these *S*‐curves as observed on the samples that have undergone milling of 5, 10, and 15 h in terms of the low values of coercivity and remanence, which would be an indication that is directed toward superparamagnetism. The coercive fields improve significantly as milling time increases. This is related with smaller crystallite sizes, indicating a transition from single‐domain to multidomain activity. Bulk NiFe_2_O_4_ ferrite is expected to exhibit modest magnetization due to net magnetic moments in the A and B sublattices. A general rise in magnetism with reducing crystallites can be explained by Ni ion migration from A to B sites (reducing Fe ions at B sites).

**FIGURE 7:**
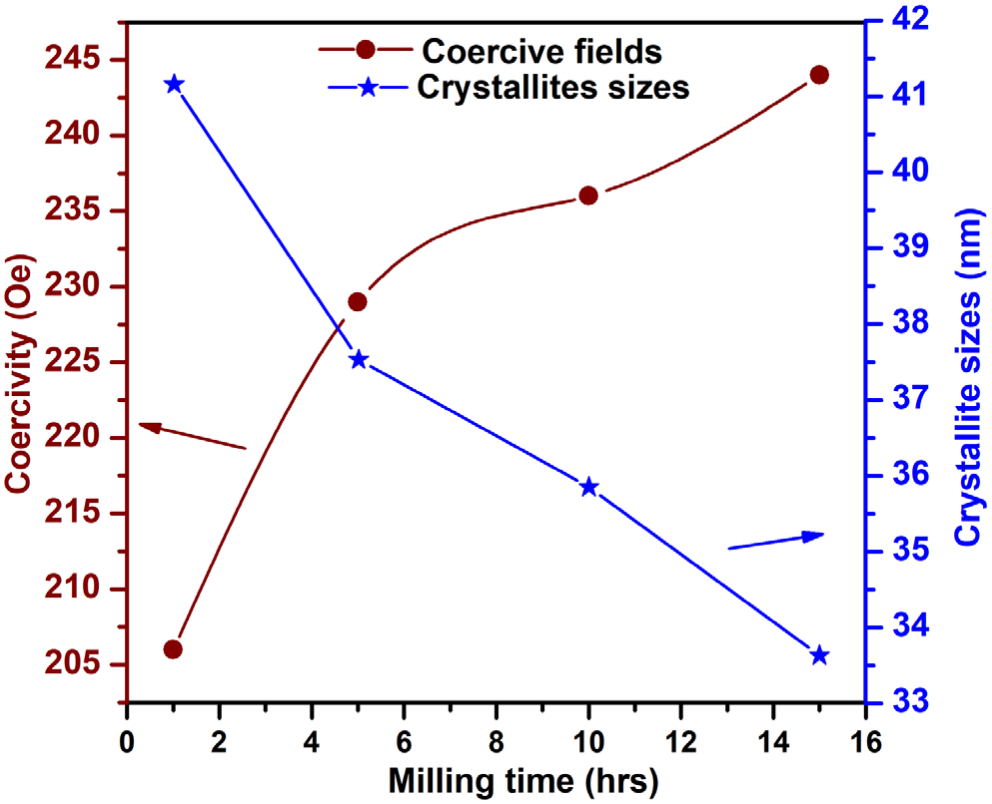
Dependence of the coercive field and crystallite and on the ball‐milling time for NiFe_2_O_4_ nanoferrites.

Improved crystallinity and better redistribution of cations to A‐ and B‐sites in the spinel lattice explain the growth of saturation magnetization (*M*_s_) to 34.1 emu/g with longer milling times (compared with 33.4 emu/g of 10 h milling). LAS fitting was used, and the results were determined. This redistribution can improve A–B superexchange interactions, facilitating a more rigid parallel spin structure and reduced spin canting at the grain boundaries [41]. Figure 7 shows the variation of coercivity and crystallite size with milling time, revealing an inverse relationship where an increase in milling time results in an increase in coercive fields and a decrease in crystallite size. This inverse trend indicates that extended milling refines the particle size, leading to enhanced surface anisotropy and increased pinning of magnetic moments. As the crystallite size approaches the single‐domain regime, domain wall motion is suppressed, resulting in higher coercivity. The crystallite sizes remain relatively large (33–41 nm) (see Table 1) and places the particles near the single‐domain/multidomain boundary for NiFe_2_O_4_. The observed behavior highlights the critical role of microstructural control in tuning the magnetic hardness of NiFe_2_O_4_ nanoparticles.

**FIGURE 8:**
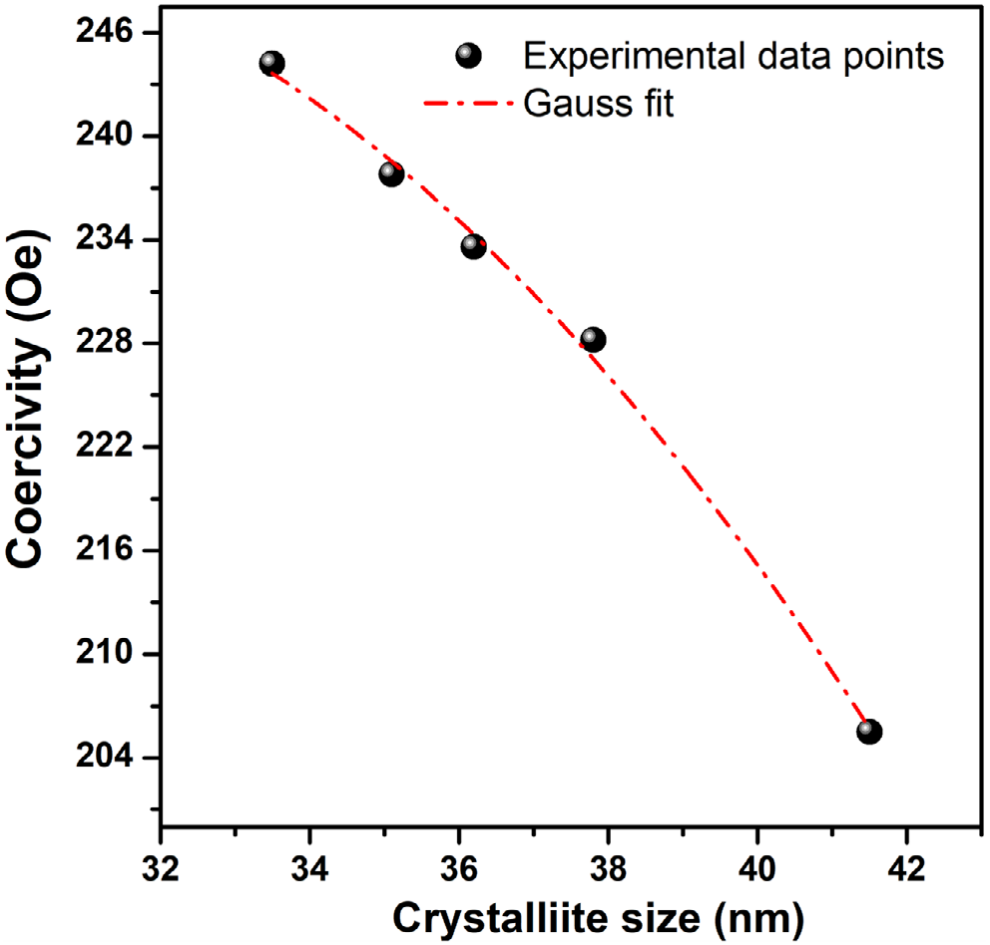
Fitted coercivity versus crystallite size curve for NiFe_2_O_4_ nanoferrites.

In Figure 8, it can be observed that *H*_c_ follows a Gaussian‐shaped dependence with a maximum of ≈245.5 Oe. The fitted curve peaks at a critical particle size (*D*_0_) of 31.1 nm, with a transition width (*w*) of 19.9 nm. This peak represents the threshold between single‐domain and multidomain behavior, beyond which coercivity decreases due to the formation of domain walls [42]. The fitted parameters confirm that magnetic moments are strongly pinned in the single‐domain regime, resulting in high coercivity values and reduced thermal fluctuation effects.

The magnetic anisotropy constant *K*_1_ was recalculated using the corrected expression derived from the LAS, given by [43]:

(7) $$K_{1}=\mu_{0}M_{S}\sqrt{\frac{105b}{8}}$$

where *μ*_0_ is the vacuum permeability (4*π* × 10^−7^ T·m/A), *M*_s_ is the saturation magnetization in A/m, and *b* is the fitting parameter obtained from the LAS high‐field fit. The expression is more effective in explaining the anisotropy contributions of nanoscale ferrites than previously simplified quadratic expressions. The *K*_1_ values calculated range between 9.528 × 10^−3^ J/m^3^ (1 h) to 5.317 × 10^−2^ J/m^3^ (15 h), which shows a gradual growth of the anisotropic strength under continuous milling. Such a trend indicates the improvement of spin–lattice coupling with the ages of increasing impact of microstructural strain and surface anisotropy with smaller crystallite sizes. Although these values are relatively low, as expected in soft ferrites, they are strong affirmations that the anisotropy can be hugely affected by small structural rearrangements of nanosized systems. To quantify magnetic anisotropy, the anisotropy constant (*K*_1_) was calculated using the relation in Equation (6) The calculated *K*_1_ values closely mirror the behavior of *M*_s_, increasing from 3.24 × 10^−6^ J/m^3^ for the disordered 5 h sample to 7.90 × 10^−6^ and 7.76 × 10^−6^ J/ m^3^ for the 10 and 15 h samples, respectively. The 1 h sample shows a slightly higher value (8.91 × 10^−6^ J/m^3^), suggesting an incomplete cationic mixing in its early milling stage.

An empirical relationship which gives a qualitative estimate of the effective domain wall energy density *γ* was used to estimate the value of this quantity [43]:

(8) $$\gamma \propto \sqrt{K_{1}}\cdot M_{S}$$

where *γ* scales with the product of anisotropy and magnetization, serving as a proxy for the energy required to propagate domain walls. In more crystalline, well‐milled samples, domain wall energy rises rapidly; at 15 h, the energy is 7.63 a.u., and an increase in energy implies an increase in domain stability and a greater resistance to magnetization reversal. This tendency helps in the suppression of non‐coherent reversal mechanisms, such as incoherent rotation and curling, in favor of coherent switching or Bloch‐type wall movement [42].

The magnetic moment per formula unit *n*_B_ was calculated using the expression [41]:

(9) $$\eta_{B}=\frac{M_{s}\cdot M_{w}}{5585}$$

where *M*_w_ = 234.38 g/mol is the molar mass of NiFe_2_O_4,_ and the factor 5585 arises from the unit conversion to Bohr magnetons. The values go up from 0.48 *µ*_B_ (1 h) to 1.39 *µ*_B_ (15 h), as one would expect, better ferrimagnetic ordering with more efficient redistribution of the cations and with the suppression of the spin disorder at the grain boundaries [40]. These values are close to those of highly crystalline bulk spinel ferrites, which means that long milling periods can repair magnetic symmetry and coherence.

### 3.4 Electron Spin Resonance Results

**FIGURE 9:**
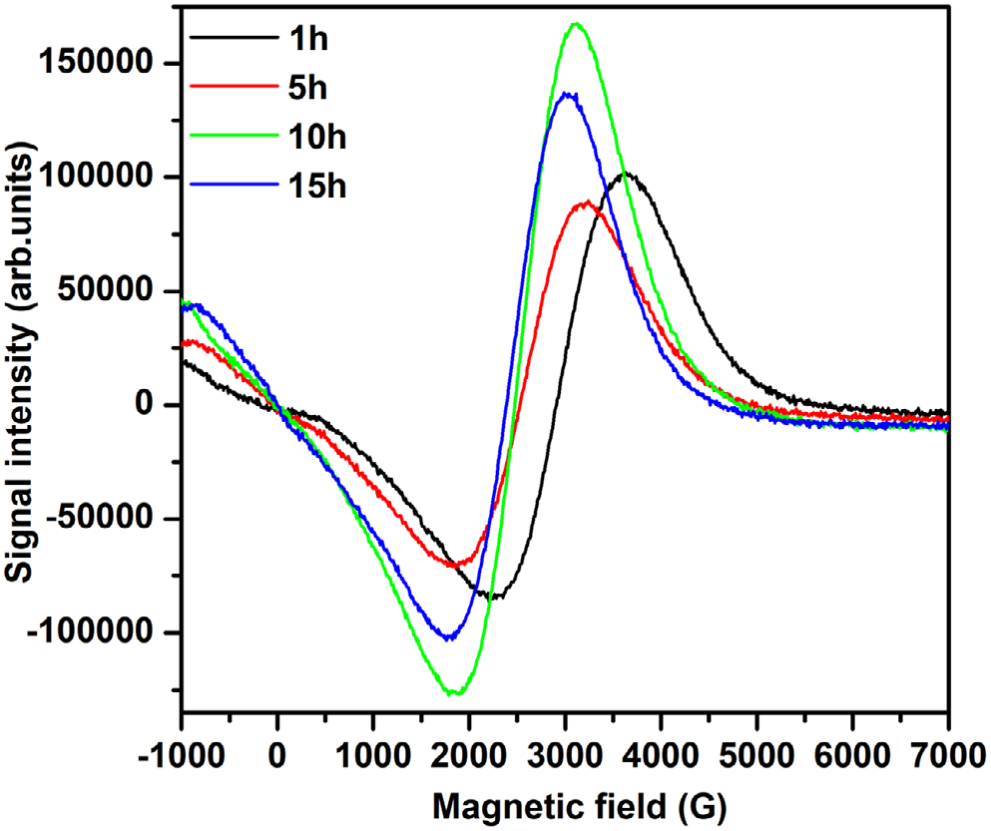
The electron spin resonance spectra as function of milling time for NiFe_2_O_4_ nanoferrites.

**FIGURE 10:**
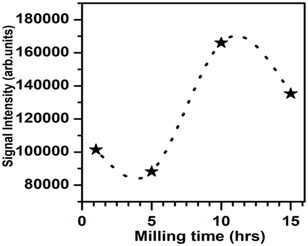
The variation of signal intensity with milling time for NFe_2_O_4_ nanoferrites.

The ESR spectra of ferrites are useful for studying the magnetic properties of magnetic materials at high frequencies because the resonance is caused by the interaction of spin and electromagnetic waves. ESR measurements on all samples were performed to better understand the information about spin‐related phenomena. The ESR spectra for NiFe_2_O_4_ as a function of milling time are presented in Figure 9. All samples’ spectra displayed a single broad line demonstrating the creation of a cubic spinel structure, which is established by XRD. The narrow ESR signal is due to uniformly highly ordered magnetic moments originating from magnetic Ni^2+^ ion. Figure 10 depicts the variation in ESR signal intensity as a function of milling time**.** The intensity fluctuates with an increasing milling time. The fluctuation is associated with the redistribution of Ni^2+^ and Fe^3+^ ions on tetrahedral and octahedral sites as result of particle sizes. This redistribution causes the magnetic moments of the sub‐lattices to be unstable. Lande's *g*‐factor is 2.0023 for a free electron; however, it is different in a solid compound [44, 45]. The ESR parameters such as resonance field (*H*_r_), *g*‐factor, line width (Δ*H*_pp_), and sin–spin relaxation time (*τ*_2_) are calculated using the relations given in our previous report [46]:

(10) $$g=\frac{hv}{\mu_{B}H_{r}}\;\mathrm{and}\;\text{Δ}H_{1/2}=\sqrt{3}\text{Δ}H_{\mathrm{pp}}$$

The spin–spin (*τ*_1_) and spin–lattice (*τ*_2_) relaxation times, were calculated using the expressions [47, 48]:

(11) $$\tau_{1}=\frac{\hbar }{g\mu_{B}H_{{\mathrm{pp}}_{1/2}}}\mathrm{and}\tau_{2}=\frac{\tau_{1}}{8}{\left(\frac{\text{Δ}H_{1/2}}{H_{r}}\right)}^{2}$$

**FIGURE 11:**
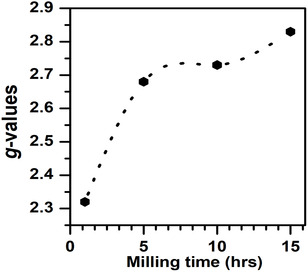
The dependence of *g*‐values on milling time for NFe_2_O_4_ nanoferrites.

In these equations, *h* is Plank's constant (6.626 × 10^−34^ J·s), “*υ*” denotes the ESR operating frequency (9.45 × GHz), “*μ*_B_” is the Bohr magneton (9.27 × 10^−24^ J/T), and (Δ*H*_pp_) is the line width. The calculated parameters are tabulated in Table 3. The dependence of the obtained *g*‐values with of milling time are also presented in Figure 11. From the table, there is a general increase in *g‐*value from 2.32 to 2.83 with an increase in milling time. *H*_r_ varies between 2385 and 2908 Oe. It can also be observed that *H*_r_ decreases with an increase in milling time. This is associated with an increase in net magnetic moments leading to an increase in super exchange interactions. The low *g*–values and line width indicate the increase in superexchange interactions and decrease in dipolar interactions.

**TABLE 3:** Resonance field (*H*_r_) *g*‐values, peak‐to‐peak line width (Δ*H*_pp_), spin–spin (*τ*_1_), and spin–lattice (*τ*_2_) relaxation times NiFe_2_O_4_ nanoferrites.

Milling time*,* h	*H*_r_, (Oe)	*g*‐value	Δ*H*_pp__12_, (0e)	*τ*_1_, ×10^−11^ s	*τ*_2_, ×10^−12^ s
	±0.3	±0.02	±4	±0.01	±0.02
1	2908	2.32	2360	2.07	1.708
5	2524	2.68	2634	1.61	1.195
10	2473	2.73	2488	1.67	2.113
15	2385	2.83	2424	1.66	2.138

The relaxation time increases with milling time for NiFe_2_O_4_ samples under investigation. This is associated with the increase in energy transfer spin system to crystal lattice, due to the reduction in particle sizes caused by milling. Table 3 also depicts that the spin–lattice relaxation time (*τ*_2_) is dependent on the spin–spin relaxation time (*τ*_1_). The 1 h milled sample revealed that *τ*_2_ is 1.708 × 10^−12^ s, which increases to a maximum of 2.138 × 10^−12^ s for the 15 h milled sample. The increase in *τ*_2_ can be credited to the stronger magnetic interactions produced by milling. Reducing crystallite size reduces phase coherence among spins, resulting in a decrease in *τ*_1_ as milling time increases.

## 4 Conclusions

Nanocrystalline spinel NiFe_2_O_4_ ferrites have been successfully synthesized using high‐energy ball mill. XRD reveals that milling for shorter period results in unreacted NiO and Fe_2_O_3_. Longer milling resulted in the formation of the cubic spinel with *Fd3* *m* space group, which influence the magnetic and electron spin resonance properties. The decrease in crystallite sizes with longer milling time shows the significance of milling on crystallite sizes. The SEM images also revealed the decrease in grain sizes with milling time. The nature of the *M* *–* *H* loops for all the samples shows soft ferromagnetic behavior. Magnetization was improved upon increasing the milling time from 1 to 15 h. Based on our data, the samples demonstrate soft ferrimagnetism with features approaching superparamagnetic‐like behavior. The increasing trend of saturation magnetization with increasing milling time is associated with the decreasing crystallite sizes. Th ESR revealed the increasing *g*‐values because of reduced resonance field. The increase in spin–spin (*τ*_1_) decreased while the spin–lattice (*τ*_2_) relaxation times increased. These were attributed to the stronger magnetic interactions due to milling time.

## Author Contributions

**Sanele Dlamini**, **Sizwe Masuku**, and **Lebogang Kotsedi** were responsible for methodology, data acquiring, analysis and drafting original manuscript. **Mohd Sajid Ali**, **Hamad A. Al-Lohedan**, **Gulam Rabbani**, **Teboho Mokoena**, **Tebogo Mahule**, **Teboho Mokhena**, **Mohd. Hashim** and **Justice Msomi** were responsible for data validation, investigation, manuscript—review and editing. **Amos Nhlapo** was responsible for conceptualization, project administration, investigation, data validation, manuscript review and editing.

## Conflicts of Interest

The authors declare no conflicts of interest.

## Data Availability

The data that support the findings of this study are available from the corresponding author upon reasonable request.
